# Use of Conventional Phototherapy vs Light-Emitting Diode for the Treatment of Unconjugated Hyperbilirubinemia in Neonates: A Randomized Controlled Trial

**DOI:** 10.7759/cureus.103208

**Published:** 2026-02-08

**Authors:** Muhammad Ijaz, Fawad Ahmed, Muhammad Shahbaz, Hammad Ahmed, Sehreen Ashraf, Hailing Ding

**Affiliations:** 1 Faculty of Medicine, Qilu Institute of Technology, Jinan, CHN; 2 Department of Paediatrics, King Edward Medical University, Lahore, PAK; 3 Department of Radiology, Qilu Hospital of Shandong University, Jinan, CHN; 4 Department of Pharmacy, Sialkot Institute of Science and Technology, Sialkot, PAK; 5 Department of Biochemistry, University of the Punjab, Lahore, PAK

**Keywords:** conventional therapy, light-emitting diode (led), neonates, phototherapy, unconjugated hyperbilirubinemia

## Abstract

Neonatal hyperbilirubinemia is a disease found in newborns, and it can lead to permanent brain damage. Unconjugated hyperbilirubinemia could be treated by using various phototherapy techniques. This study sought to compare the mean duration of treatment required with light-emitting diode (LED) versus conventional phototherapy in newborns with unconjugated hyperbilirubinemia. The investigation included 452 patients, randomly distributed into two groups. LED phototherapies were administered to Group 1, while conventional phototherapy was administered to Group 2. Total serum bilirubin levels, along with unconjugated serum bilirubin levels, were checked at baseline. Once the serum total bilirubin level fell below the cutoff level, phototherapy was stopped. The total duration of phototherapy, in hours, along with other demographic data, was recorded and subsequently analyzed using a Student’s t-test. In Group 1, the mean gestational age was 37.51 ± 1.02 weeks, whereas in Group 2, it was 37.54 ± 1.02 weeks. The mean duration required for treatment with LED (Group 1) versus the conventional method of phototherapy (Group 2) in neonates with unconjugated jaundice was 36.26 ± 3.24 hours vs 45.5 ± 5.13 hours (p<0.0001), respectively. It was concluded that the mean duration required for the treatment of newborns affected by unconjugated hyperbilirubinemia was significantly lower with LED than with conventional phototherapy.

## Introduction

Hyperbilirubinemia is reported to be the most common condition in newborns. It has been reported that 60% of term babies and 80% of preterm babies show signs of hyperbilirubinemia within the first week of their lives [1]. In newborns, total serum bilirubin levels that are too high can cause severe and permanent brain damage. The main side effect of hyperbilirubinemia is bilirubin-induced encephalopathy, which happens when unconjugated bilirubin attaches to brain tissue once it has crossed the blood-brain barrier, since the blood-brain barrier of the infant is not well developed [2]. One of the neurological side effects of bilirubin on the brain is acute bilirubin encephalopathy (ABE), while kernicterus, which is a persistent condition that results in permanent impairment, is another [3]. The two primary treatment approaches to prevent bilirubin-induced brain damage in newborn jaundice are phototherapy and exchange blood transfusion. The type of treatment depends on the total serum bilirubin level; however, phototherapy is the most common option [4]. Compared to the risks associated with exchange transfusion, phototherapy is a less risky option.

According to the American Academy of Paediatrics (AAP)'s most recent recommendations, intense phototherapy involves the application of blue light (bands of 430-490 nm) at 30.0 microwatts/cm^2^/nm or greater to as much of the body as feasible [5]. Traditional phototherapy methods include the use of fluorescent tubes, halogen lamps, and fibre-optic blankets. More recently, Light-Emitting Diodes (LEDs), which have a significantly higher radiant energy compared to older sources, can also be used for phototherapy [6].

There are some significant drawbacks to using traditional light sources for phototherapy. Conventional light sources generate a significant amount of heat and should not be placed too close to a baby. Fibreoptic blankets can be used to remedy this issue; however, due to their small exposure area, they are not very successful in the field [7]. LEDs have lately been researched as potential replacements in phototherapy systems due to these constraints. LEDs generate little heat, allowing them to be placed quite close to the baby. When compared to conventional light sources, LEDs have an extended lifespan (averaging 20,000 hours) and consume less energy [8]. This reduces their price and they provide a high-intensity, monochromatic light featuring a limited spectral band and a special wavelength of 458 nm, which falls within the bilirubin absorption spectrum [9]. The benefits of LED phototherapy, including reduced heat, low energy consumption, and a longer half-life, are well known. In the South Asian population, there are very few available reports comparing LED phototherapy with conventional phototherapy in late preterm and full-term infants [10].

In this study, we will compare the duration required for treatment with conventional phototherapy vs LED phototherapy in late preterm and full-term neonates with unconjugated hyperbilirubinemia.

## Materials and methods

Research rationale and study background

The study was a randomized controlled experiment conducted in the Pediatric Unit 1 and the Neonatology Unit at King Edward Medical University/Mayo Hospital, Lahore, Pakistan. The study was conducted over six months. The Institutional Review Board and Ethical Committee of King Edward Medical University, Lahore, approved this study (No. 607/RC/KEMU dated 07/09/2021), and the College of Physicians and Surgeons of Pakistan (CPSP) approved vide reference no. CPSP/REU/PED-2019-066-5374 dated 15/12/2020.

Sample size

The study was conducted on a cumulative 452 cases, with 226 cases in each group. The sample size was estimated using a treatment interval of 37.5 ± 26.8 hours for LED and 45.3 ± 32.1 hours for conventional phototherapy [11]. A 95% confidence interval (CI) was selected, and the study’s power was set to 80% to determine the sample size. Probability random sampling was employed in the study.

Data collection methodology

Inclusion criteria for this study were neonates having jaundice; age more than or equal to two days and less than 15 days; gestational age equal to or more than 35 weeks; with serum bilirubin levels above the cut-off level developed by the AAP [12]. Exclusion criteria were neonates having serum bilirubin levels above the cut-off for exchange transfusion as recommended by the AAP chart, blood group incompatibilities, jaundice that appears within 24 hours following birth, culture-positive or clinical sepsis, positive direct Coombs test, requiring mechanical ventilation and ionotropic support, moribund, or critically ill neonates.

Parents of the 452 patients included in the trial were briefed on the study objectives, and their written informed consent was taken before administering any of the research protocols (all relevant data have been deposited in the CPSP repository and can be accessed upon request). A detailed history and clinical examination of patients meeting the inclusion criteria were carefully recorded. Demographic details, including name, gestational age at birth, gender, and birth weight, were recorded. The blood group, complete blood count (CBC) with peripheral blood film, direct Coombs’ test, reticulocyte count, and serum bilirubin level (total, direct, and indirect) were sent to the hospital's pathology lab.

Outcome measures

By using the lottery approach, the patients were randomized into two groups. Randomization was performed using a simple randomization technique. A random allocation sequence was generated prior to patient enrollment using a computer-generated random number table. Eligible patients were assigned to either Group 1 (LED phototherapy, irradiance 35 μW/cm²/nm) or Group 2 (conventional phototherapy, irradiance 22 μW/cm²/nm) in a 1:1 ratio. Allocation concealment was ensured using sequentially numbered, opaque, sealed envelopes (SNOSE), which were prepared by a staff member not involved in patient recruitment or outcome assessment. The envelopes were opened only after the patient's enrollment, ensuring concealment of the group assignment. Irradiance was measured using a standardized irradiance meter before the initiation of therapy. These levels comply with the AAP guidelines. Total serum bilirubin levels, along with unconjugated serum bilirubin levels, were checked at baseline using an AU480 Beckman Coulter Biochemistry analyzer (Beckman Coulter, Inc., California, US). Bilirubin was checked by a transcutaneous bilirubinometer every four to six hours till the end of the treatment. Serum bilirubin levels were re-measured using the same analyzer at the end of treatment. Laboratory staff responsible for measuring serum bilirubin levels were blinded to group allocation. The rationale for blinding the laboratory staff was to minimize the risk of measurement and analytical bias, as serum bilirubin levels constituted the primary outcome of the study. The skin response and dehydration caused by phototherapy were monitored in the infants. Phototherapy was stopped once the serum total bilirubin level was below the cut-off level for phototherapy in both groups, as specified by the AAP chart. AAP nomograms were applied uniformly to all enrolled neonates, taking into account the gestational age and the postnatal age (in hours), as recommended by the AAP guidelines. Six hours after ceasing phototherapy, blood bilirubin levels were measured. The total duration of phototherapy (in hours), along with other demographic data, was recorded and subsequently analyzed.

Statistical analysis

For the statistical study, IBM SPSS Statistics for Windows, Version 23 (Released 2015; IBM Corp., Armonk, New York, United States) was used. Age, birth weight, serum bilirubin levels, and treatment duration (in hours) were all quantitative variables that were provided as means and standard deviations. Gender and gestational age were two qualitative variables that were expressed as frequencies and percentages. The student’s t-test was employed to compare the lengths of therapy in the two groups. Gender, age, gestational age, and birth weight were used to stratify the data. Furthermore, the post-stratification t-test was used, with a p-value of <0.05 considered significant.

## Results

Demographic characteristics of the study population

**Figure 1:**
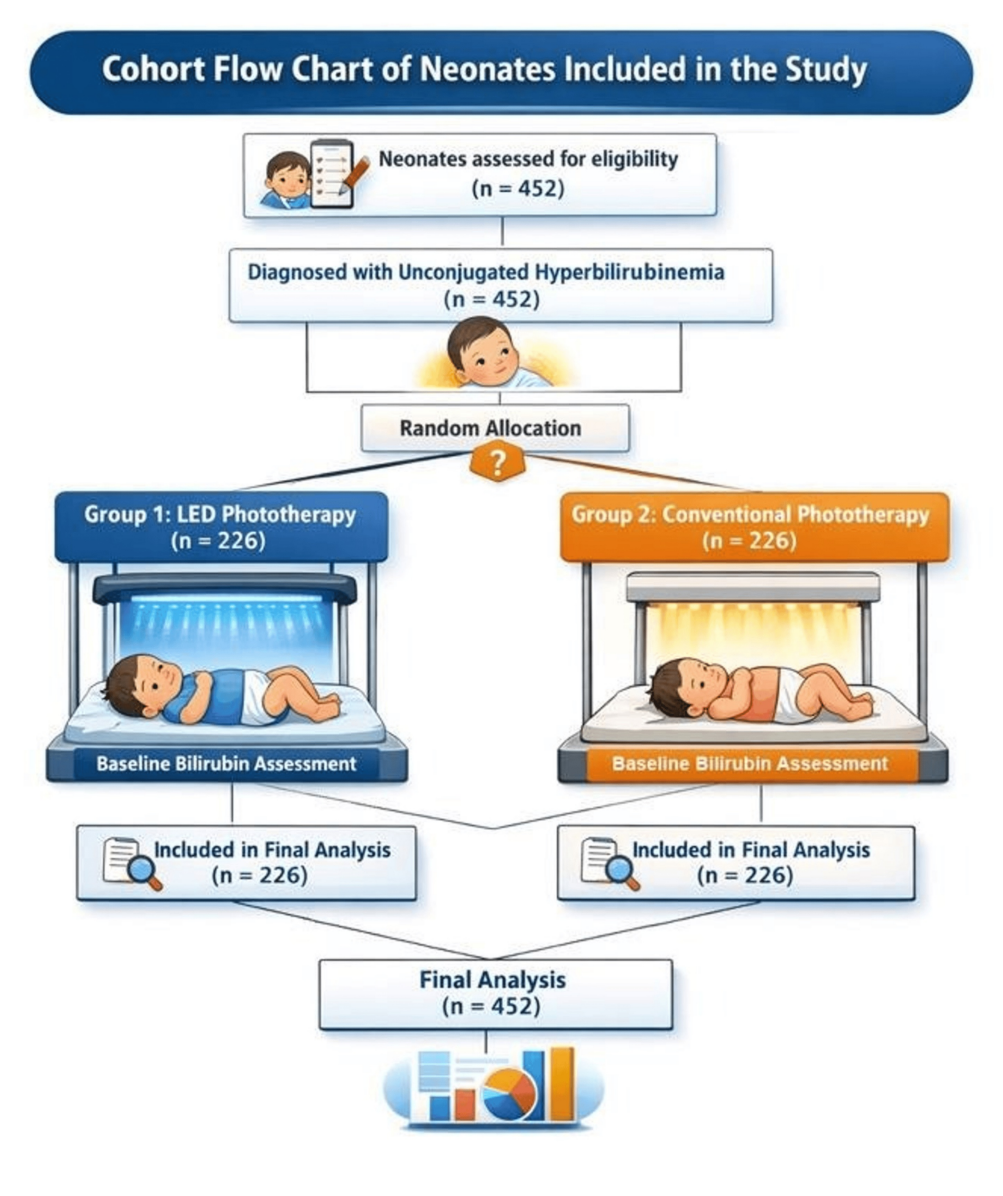
Cohort flow chart illustrating the patient selection process Image credit: Created by authors using Adobe Photoshop (Adobe Inc., California, US) and Canva (Perth, Australia).

Cumulatively, 452 cases (226 cases per group) fulfilling the eligibility criteria were assessed to evaluate the specificity of treatment with LED versus conventional phototherapeutic treatment in neonates diagnosed with unconjugated hyperbilirubinemia (Figure 1). 

The age distribution showed that 76.55% neonates (n=173) in Group 1 and 80.09% (n=181) in Group 2 fell between two to 10 days of life; however, 23.45% (n=53) in Group 1 and 19.91% (n=45) in Group 2 fell between 11-14 days of life. The mean age of infants was 8.43 ± 2.80 days in Group 1 and 8.24 ± 2.78 days in Group 2 (Table 1).

**Table 1:** Demographic characteristics and baseline clinical variables of the study population (n=452) Group 1: LED phototherapies were administered; Group 2: conventional phototherapy was administered. The results were found to be significant as P value was <0.05.

Variable	Group 1 (n = 226)	Group 2 (n = 226)	P Value
Age (days)	n (%)	n (%)	
2–10	173 (76.55)	181 (80.09)	0.03
11–14	53 (23.45)	45 (19.91)	0.03
Gender	n (%)	n (%)	
Male	138 (61.06)	145 (64.16)	0.03
Female	88 (38.94)	81 (35.84)	0.03
Birth weight (g), mean ± SD	2574.43 ± 145.86	2569.78 ± 155.72	0.008
Gestational age (weeks), mean ± SD	37.51 ± 1.02	37.54 ± 1.03	0.009
Baseline bilirubin (mg/dL), mean ± SD	12.35 ± 1.34	12.42 ± 1.16	0.006
Duration of treatment (hours), mean ± SD	36.26 ± 3.24	45.50 ± 5.13	0.003

The gender distribution revealed that 61.06% (n=138) in Group 1 and 64.16% (n=145) in Group 2 were male participants, whereas the female population comprised 38.94% (n=88) of Group 1 and 35.84% (n=81) of Group 2 (Table 1).

Baseline clinical characteristics and comparison of treatment duration

As shown in Table 1, the mean birth weights were 2574.43 ± 145.86 grams in Group 1 and 2569.78 ± 155.72 grams in Group 2. The mean gestational age of infants in Group 1 was 37.51 ± 1.02 weeks, whereas that in Group 2 was 37.54 ± 1.03 weeks. The mean bilirubin level at baseline in both groups was evaluated and recorded as 12.35 ± 1.34 mg/dL in Group 1 and 12.42 ± 1.16 mg/dL in Group 2. A comparison of the mean duration required for treatment with LED versus conventional neonatal phototherapy for unconjugated hyperbilirubinemia was 36.26 ± 3.24 hours in Group 1 and 45.5 ± 5.13 hours in Group 2 (Table 1). This difference was statistically significant (p=0.003), indicating that LED phototherapy reduced the time required to reach safe bilirubin levels compared to conventional phototherapy.

Stratified analysis of treatment duration across demographic and clinical variables

Gender, age, gestational age, and birth weight were used to stratify the data. The post stratification T-test was used, with a p-value of not greater than 0.05 being considered significant (Table 2).

**Table 2:** Stratified analysis of treatment duration by age, gender, gestational age, and birth weight Group 1: LED phototherapies were administered; Group 2: conventional phototherapy was administered; Data are presented as mean ± SD; n denotes the number of participants in each stratified subgroup.

Variables	Categories	Group 1 (n)	Group 2 (n)
Age (in days)	2–10	36.10 ± 3.28 (n=173)	45.37 ± 5.15 (n=181)
	11–14	36.77 ± 3.09 (n=53)	46.02 ± 5.05 (n=45)
Gender	Male	36.34 ± 3.19 (n=138)	45.26 ± 5.35 (n=145)
	Female	36.14 ± 3.33 (n=88)	45.93 ± 4.70 (n=81)
Gestational age (weeks)	36–37	35.92 ± 3.23 (n=117)	45.42 ± 4.96 (n=110)
	>37	36.62 ± 3.23 (n=109)	45.58 ± 5.30 (n=116)
Birth weight (grams)	≤2500	36.36 ± 3.17 (n=77)	46.30 ± 5.01 (n=83)
	>2500	36.21 ± 3.28 (n=149)	45.03 ± 5.15 (n=143)

Results revealed that, in both age groups (two to 10 days and 11-14 days), neonates receiving LED phototherapy consistently required significantly shorter treatment durations than those receiving conventional phototherapy (p=0.0001). Both male and female infants treated with LED phototherapy showed shorter treatment times compared to their counterparts in the conventional group, again with strong statistical significance (p=0.0001). Whether infants were 36-37 weeks or more than 37 weeks of gestation, LED phototherapy remained superior in reducing treatment duration. Similarly, in neonates weighing ≤2500 g and >2500 g, LED phototherapy consistently resulted in faster resolution of hyperbilirubinemia (Table 2).

These stratified results confirm that the benefit of LED phototherapy was robust and independent of age, gender, gestational age, or birth weight.

## Discussion

In traditional phototherapy, compact fluorescent lamps (CFLs) or halogen lights are utilized. LEDs, reported to have exceptional features such as lower heat production, portability, power efficiency, and resilience, are usually applied as the light source for phototherapy [13]. An insufficient number of studies have been conducted to compare the effectiveness of conventional and LED phototherapy, and the results have been inconclusive. Nonetheless, a Cochrane review reported that both phototherapy modalities have a similar efficacy in reducing total serum bilirubin levels [14], which is consistent with our findings, as both groups achieved comparable bilirubin reduction to levels below the AAP treatment thresholds. Infants receiving LED phototherapy reached the phototherapy discontinuation threshold in a shorter time, leading to reduced treatment duration, while achieving similar bilirubin control.

In Pakistan, there are very few reports comparing LED phototherapy with conventional phototherapy in late preterm and full-term infants. In this study, we compared the duration required for treatment with LED phototherapy vs conventional phototherapy in late preterm and full-term neonates with unconjugated hyperbilirubinemia. In our study, the demographics between the two groups were not significantly different.

A comparison of the mean duration required for treatment with LED versus conventional neonatal phototherapy with unconjugated jaundice showed 36.26 ± 3.24 hours in Group 1 and 45.5 ± 5.13 hours in Group 2, suggesting a significant difference between the two groups (p=0.003). Our findings were confirmed by a previous study, which revealed that the duration of treatment with LED phototherapy was 37.5 ± 26.8 hours, in contrast to 45.3 ± 32.1 hours with conventional phototherapy [15]. Another trial [14] compared the efficiency of conventional phototherapy and LED phototherapy in reducing total serum bilirubin levels, and the latter approach was more potent than the former. Furthermore, patients who received LED therapy had elevated levels of urine bilirubin. Both therapies were reported to be comparable, with few side effects.

Another study investigated various parameters, including total serum bilirubin reduction and the mean interval for treating unconjugated neonatal jaundice using LED and conventional phototherapy [16]. Their findings revealed that 55% were male subjects, while 45% were female subjects. The mean duration of LED therapy was found to be lower with a p-value of 0.0001, and the reduction in total serum bilirubin was found to be faster with LED therapy as compared to older treatments. It was also reported that patients who received LED treatment had a lower degree of hyperthermia [17]. Therefore, it was concluded that LED therapy is more efficient than conventional therapy. The observed advantage of LED phototherapy pertains solely to treatment efficiency as reflected by the shorter duration of phototherapy.

Limitations of the study

This study has certain limitations that need to be acknowledged. First, the primary outcome focused on the duration of phototherapy, while other clinically relevant outcomes, such as the decline rate of bilirubin, post-phototherapy rebound hyperbilirubinemia, and extended neurological follow-up, were not assessed. Second, although randomization was performed, the study was conducted at a single center, which may limit the generalizability of the findings to other settings with different patient populations or phototherapy practices. Third, blinding was not feasible due to the clearly distinguishable nature of the phototherapy devices, which may have resulted in performance bias. Despite these limitations, this study provides valuable evidence supporting the effectiveness of LED phototherapy in reducing treatment duration in neonates with unconjugated hyperbilirubinemia.

## Conclusions

In conclusion, LED phototherapy is a more efficient treatment option than conventional phototherapy for neonates with unconjugated hyperbilirubinemia. Newborns treated with LED phototherapy required a significantly shorter treatment duration to achieve safe bilirubin levels, despite comparable baseline characteristics. The consistent reduction in treatment time across different ages, genders, gestational ages, and birth weights highlights the reliability of LED therapy. Given its shorter treatment duration, LED phototherapy could be considered the preferred modality for managing unconjugated hyperbilirubinemia in late preterm and term neonates.

## Figures and Tables

**Figure 2:**
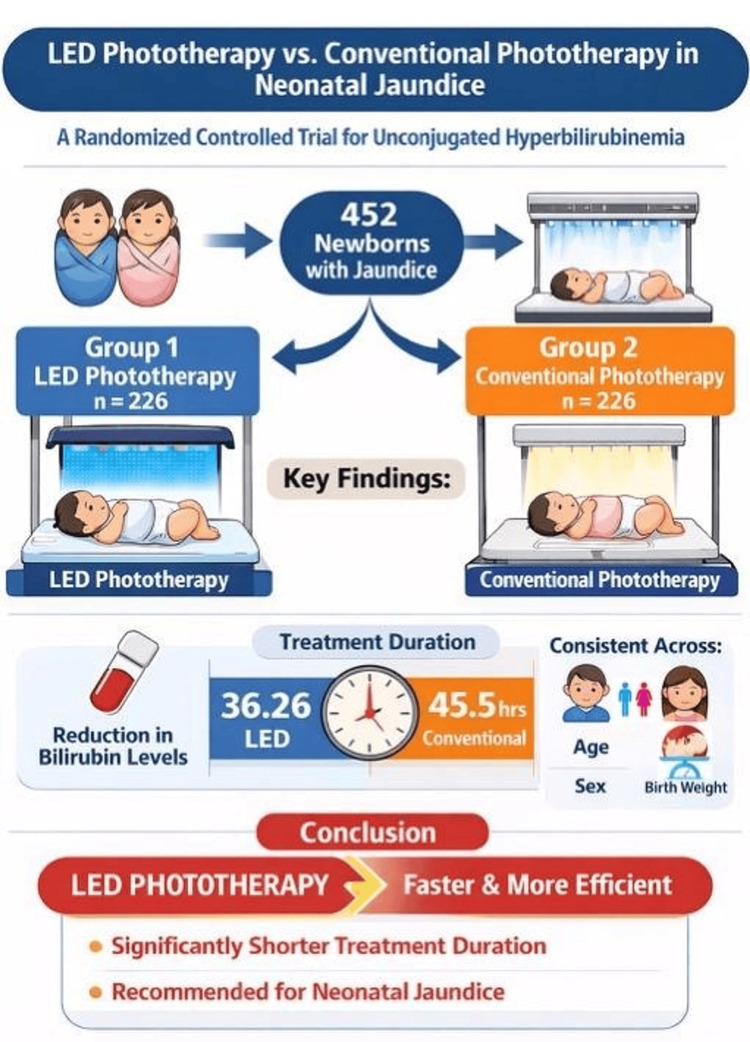
Graphical abstract Image credit: Created by authors using Adobe Photoshop (Adobe Inc., California, US) and Canva (Perth, Australia).
